# Social Interaction and Collaboration among Oncology Nurses

**DOI:** 10.1155/2015/248067

**Published:** 2015-05-31

**Authors:** Jane Moore, Dawn Prentice, Maurene McQuestion

**Affiliations:** ^1^Department of Nursing, Faculty of Applied Health Sciences, Brock University, 500 Glenridge Avenue, Saint Catharines, ON, Canada L2S 3A1; ^2^Princess Margaret Cancer Centre, University Health Network, 610 University Avenue, Toronto, ON, Canada M5G 2M9

## Abstract

Collaboration is a complex process influenced by organizational, professional, interpersonal, and personal factors. Research has demonstrated that collaboration may also be influenced by social factors. Nurses spend much of their time working in collaborative teams, yet little is known about how they socially interact in practice. This qualitative case study explored nurse perceptions of social interaction in relation to collaboration. Data were collected using telephone interviews and documentary reviews from fourteen oncology nurses employed at one cancer center in Canada. Thematic analysis revealed two themes: *knowing you is trusting you* and *formal and informal opportunities*. Nurses reported that social interaction meant getting to know someone personally as well as professionally. Social interaction was enacted inside of work during breaks/meals and outside of work at planned events. Social interaction was facilitated by having a long-term current and/or previous professional and personal relationship. The barriers to social interaction included a lack of time to get to know each other, workload issues, and poor interpersonal skills. Findings suggest that social interaction is an important factor in the collaborative relationship among oncology nurses. Nurse leaders need to promote social interaction opportunities and facilitate educational sessions to improve social and interpersonal skills.

## 1. Introduction

Collaboration has been described in the context of healthcare as a complex process by which interdependent professionals work together to provide patient care [1]. Studies have shown that effective collaborative teamwork is associated with higher levels of personal and professional satisfaction, healthier workplaces, and lower levels of stress [2, 3]. Several factors can influence collaboration including those at the individual and organizational level. Individual factors include a willingness to collaborate, the desire to achieve a common goal, possessing effective communication skills, and having mutual trust and respect for each other's professional contributions [4]. At the organizational level, it is important to have leadership that supports collaborative practice by providing resources such as time and space for individuals to develop and maintain interpersonal relationships needed for collaboration [5].

In addition to individual and organizational factors, social interaction has been found to influence collaboration among healthcare providers. Social interaction is described as a process whereby information is exchanged between individuals involved in a friendly relationship [6]. Research has shown that individuals require some form of social interaction in order to successfully engage in collaborative practice [7]. San Martín-Rodríguez et al. [8] suggested that the interactional determinants willingness to collaborate, effective communication skills, and the existence of mutual respect and trust contributed to successful interprofessional collaboration. Pullon [9] reported that socializing at work by displaying humor with members of the interprofessional team positively contributed to the development of trust, respect, and collaboration between nurses and physicians. Similarly, several authors found that daily social exchanges contributed to the development of mutual trust and collaboration among colleagues in the education field [10, 11].

Collaboration among healthcare providers, including nurses, is an essential element in the delivery of high quality patient care and the creation of healthy work environments [8]. Collaborating effectively is a professional responsibility and competency that applies to all nurses [12]. While the influence of organizational and individual factors on collaboration among interprofessional teams has received some attention, there has been limited research to date on social interaction in relation to collaboration among nurses [13]. Given that nurses spend more time working together in collaborative relationships than any other healthcare provider, this qualitative study was undertaken to explore how nurses perceive their social interactions with each other and what factors influence social interactions in their collaborative practice. The findings of the study contributed to recommendations for nurse leaders to improve social interaction among nurses.

## 2. Methods

A qualitative methodology was chosen as it supports an interpretive approach to gaining an understanding of the phenomenon of interest (social interaction among nurses) [14]. An exploratory, descriptive* case study* [15] using an embedded, single case design was selected to provide an in-depth description of social interaction among oncology nurses within the context of collaborative practice. A key component of case study research is the use of multiple sources of evidence, a strategy which enhances data credibility [15]. Data sources for this study included individual interviews and documentary reviews. Case study is an appropriate method to use when examining contemporary phenomenon in its real life context and when addressing “how” and “what” questions [15]. For this study, one main research question was asked: how do oncology nurses perceive social interaction in relation to collaboration in ambulatory and in-patient settings at a cancer hospital? The interview guide used the following three questions to assist the researchers with gathering information on the topic. (1) How was social interaction enacted when collaborating among oncology nurses? (2) What does social interaction mean in relation to collaboration? (3) What factors influenced social interaction in relation to collaboration among oncology nurses?

The case or the main unit of analysis was oncology nurses at one cancer center (*place*), and the embedded units were the nursing roles. Purposive, maximum variation sampling was achieved by obtaining information from practicing oncology nurses working in different nursing roles and on different clinical units [14]. To be enrolled in the study participants had to meet the following criteria: they were registered nurses (RN) or nurse practitioners, employed full-time or permanent part-time in either a specialized oncology nurse or advanced oncology nurse role [16], and employed by the cancer center and working on an in-patient or ambulatory adult unit for a minimum of one year (*time*).

## 3. Data Collection

Recruitment strategies included emailing all nurses (approximately 500) employed by the cancer center as well as providing information about the study at weekly nursing staff meetings. Eligible participants were emailed an information letter and the informed consent. No incentive was offered to participants in exchange for participation in the study. The use of multiple sources of data is a principle of case study design [15]. Data sources for this study were collected over 10 months in 2013 through open-ended, semistructured telephone interviews and document reviews. Data were collected until saturation occurred and no new themes emerged.

### 3.1. Interviews

Telephone interviews are a convenient, effective method for data collection [17]. Telephone interviewing was selected as it offered a cost effective, flexible, ease of use approach to gathering data from the participants [17]. Each individual telephone interview took place during unpaid work time and lasted 45–60 minutes on a date selected by the participant. The nurses were asked to share their experiences and thoughts about social interaction in relation to collaboration and the influence of social interaction on their collaborative relationship. The interviews were audiotaped and transcribed verbatim. After the 14th interview no new information emerged and data saturation was achieved.

### 3.2. Documents

To corroborate and augment evidence obtained from the telephone interviews, a review of documents was undertaken. The documents were accessed online through public websites and were selected as they provided information on the nurses' competencies, qualifications, and professional, regulatory, organizational, and educational factors that may have influenced social interaction and collaboration. Included in the document review were (a) the nurses' job descriptions at the cancer center, (b) the Canadian Nurses Association Framework for the Practice of Registered Nurses in Canada [18], (c) the College of Nurses of Ontario National Competencies for RNs [19], and (d) the Canadian Association of Nurses in Oncology (CANO) Standards of Care, Roles in Oncology Nursing and Roles Competencies [16].

## 4. Data Analysis

A thematic analysis was conducted on the interviews and documents. Thematic analysis entails “identifying, analyzing, and reporting patterns within data” [20]. For this study, the analysis process involved each researcher listening to the audiotaped interviews and making initial notes, followed by a first reading of the transcripts. Once a second reading of the transcripts was completed the researchers developed initial codes by hand. After completion of initial coding, the researchers compared coding and reached consensus. NVivo version 10 [21] was used to organize the transcription data and highlight emerging themes. Thematic analysis of relevant documents took place using a similar procedure to that of the interview data [20]. Documents were analyzed for key themes and compared to the interview data to see if similar or different themes were captured.

## 5. Rigor

Yin's [15] principles of data collection and Lincoln and Guba's [22] criteria for establishing trustworthiness were used to ensure rigor in the study. Multiple sources of evidence were used to achieve credibility and were seen as a form of triangulation. The researchers triangulated interview and documentary data to develop a holistic and contextual portrayal and corroborate the phenomenon under study. Member checking activities were completed after emailing the participants a draft of the initial findings and requesting their comments. A study database and a chain of evidence strengthened dependability. Transferability was accomplished through careful attention when describing the methodological components of the study and triangulation strategies [22]. The researchers triangulated interview and documentary data to develop a more holistic and contextual portrayal and corroborate the phenomenon under study. Confirmability occurred by developing codes, categories, and definitions that could be utilized by other researchers.

## 6. Ethical Considerations

Approval of the study was obtained from the research ethics board at the university and the hospital where the study was conducted. Confidentiality of the participants was ensured by using individual coding numbers (e.g., RN001), and they were informed their identity would not be disclosed. Participation was voluntary and participants could withdraw from the study at any time. Written informed consent was obtained from all participants and all participants completed the study.

## 7. Results

Fourteen oncology nurses participated in the study. The demographic information can be found in Table 1. Two themes emerged from the data analysis: (1) knowing you is trusting you and (2) formal and informal opportunities.

*Theme 1: Knowing You Is Trusting You*. The first theme* knowing you is trusting you* is related to the meaning of social interaction when collaborating with oncology nurses. The majority of nurses referred to social interaction as a means of getting to know someone personally. RN002 stated:

*You have to be able to interact with nurses not only in a work environment but also at a personal level. You need to know the person a bit and understand that person…in order to collaborate with them.*

Similarly, other nurses reported that social interaction meant knowing something about each other's lives both at work and at home:

*I think social interaction is very important and knowing bits about each other and being interested in each other…helps us communicate and collaborate…. (*RN*004)*

*Its [social interaction] about a team having a common interest or a common purpose of being together that allows them to collaborate or venture off to folks that they know…socializing at work or like recently at a professional conference…talking about work or personal stuff…. (*RN*007)*

This theme also reflected the factors that influenced social interaction in relation to collaboration. The majority of nurses said that they preferred to socially interact with other nurses who they had known over a period of time and they had formed relationships with. RN008 said:

*We have worked together for a long time…day in and day out…some [RNs] have been through school together…and we have social relationships that go beyond just working together…I don't want to say the term clique, but some people are not really part of that model…like the casual staff…or new staff…they are kind of out of that…not included…we have already formed our groups…and collaborate best with them….*

Similarly, RN010 reported that social interaction contributed to a long-term relationship that was supportive when there were challenges with workload and patient care issues:

*Knowing her for over twenty years really helps when the day is crazy busy…we socialize by having common interests and it helps with the stress to talk about watching a movie or…about a news item…we don't see each other outside of work…but I consider her a normal friend…I go to her for help…and I think the relationship has built because we socialize….*

The interpersonal skills of the nurses were also considered a factor that could positively or negatively influence social interaction. Most of the nurses said they were not interested in socially interacting with nurses who had poor attitudes, negative personalities, or those they did not trust.

*I appreciate other people's points of views but if they have negative attitudes or personalities…the negative Nancy's…are difficult to get along with…I am not interested in being social [with them]…I think there needs to be more social factors that people need to deal with in order to work better together…but difficult personalities don't make me want to get to know them better…. (*RN*012)*

*Some nurses talk behind each other's back and things like that…that's not going to make me want to socialize or collaborate with them…one minute they would be smiling at me, and laughing with me, and then the next minute they would be going to talk to somebody and say how much they dislike me…it's going to be impossible to get over…because of history…this is the case in our unit…and truthfully I'm not really sure how to fix it…it's a constant problem with some nurses being really difficult…always constant conflict…. (*RN*005)*

Lastly, the majority of nurses said that they preferred to socially interact inside and outside of work with other nurses from the same age group as themselves:

*Some of them [the nurses] are closer than others…I definitely think there is a generational piece to that…they are all around the same age… they hang out together at work…and several are friends outside of work…it has created a pretty tight group…when it comes to collaborating they look to each other. (*RN*006)*

*You know people in the same age cohort…have similar interests and experiences…collaborate well because they work well together socially and professionally…they just have more in common…more to pull them together as a team…. (*RN*008)*

*The attitude of some senior staff…is not open and does not encourage social interaction…except to people they know…it's threatening to people…mainly new nurses…and when someone is threatened that puts them in a not so pleasant light…they don't want to socialize or collaborate…. (*RN*007)*

*Theme 2: Formal and Informal Opportunities*. The second theme* formal and informal opportunities* related to how social interaction was enacted among nurses when they collaborated. The nurses socially interacted at work and outside of work. At work, the nurses socially interacted during the time they provided patient care. This social interaction was unstructured, spontaneous, and informal. RN001 said:

*We only get snippets of time [to socialize]…during our shift…so we tell stories on the fly… use humour…laugh…and tell jokes…its very necessary in oncology…because it lightens the stress and creates some bond between us…that helps for collaboration.*

The nurses socially interacted at work during scheduled breaks and meals. RN013 said that the time away from the clinical unit provided her with the opportunity to get to know someone or to get to know someone better.

*I think nurses who are new to the unit, or even people that you know…you want to have coffee or lunch with them…to get to know on a more personal level and helps you in your collaboration…it can be sitting over coffee discussing something personal or discussing work related stuff…this really helps you get a better understanding of that person…where they are coming from…what knowledge they have…and this understanding of them helps collaboration at work. *

Alternatively, the nurses reported they often missed their breaks and/or meals due to patient care and other workload issues and they felt this had a detrimental effect on their collaborative relationship. RN014 said:

*We need to make sure we get our time off the unit…so that we can shoot the breeze…not only solve problems of the clinic kind of thing…but sit down and chat about life in general…I like to see pictures of her kids…things that are important to her…that helps to get to know her as a person…not just a nurse…it's good for when need to collaborate…and our work relationship.*

Social interaction among the nurses occurred at work in the form of scheduled unit, program, or professional meetings. RN002, an advanced practice nurse, reported these meetings were used as a means of connecting with nurses who they seldom saw due to working on a different shift or with nurses who they had little time to socialize with due to the demands of their clinical work:

*Because we have a lot of complications with our patient population…you have to know each other…as a person and as a nurse…this is a tough environment…you don't have much control over things…you have to understand each other's contributions…we don't see each other that often…so at these meetings…socially interacting with these people [oncology nurses] helps build these relationships.*

Some nurses socially interacted outside of work and they viewed this as important to collaboration and building and maintaining their relationship. The interactions outside of work were arranged by the nurses as a form of a social activity. RN004 said:

*We not only come in early for meetings [staff meetings] so that we can see each other…we also go out for a beer or go to dinner once in a while…we make a real effort to get together…to shoot the breeze…have a laugh…get to know each other…reconnect…socializing reinforces that we are here to together…we work together…and when times are tough at work…we support each other…and collaborate well….*

## 8. Discussion

Collaboration among oncology nurses is a complex process that involves more than just working together in close physical proximity. Our study aimed to understand how oncology nurses perceived social interaction in relation to collaboration in the practice setting. We found that social interaction was an important antecedent of collaboration, an element that must be present prior to the development of successful collaboration. Whether it is through formal or informal opportunities, social interaction among the nurses was viewed as a means of getting to know each other professionally and personally. Given that the work of nurses involves regular, close contact with one another, it is not surprising that nurses require some “social” as well as “work” interactions as these exchanges contribute to the determinants of collaboration: positive interpersonal relationships, effective communication, and mutual respect and trust [8]. The theme “knowing you is trusting you” highlighted the importance of social interaction as a means of developing and maintaining trust in the collaborative relationship. This finding aligns with research noted in the healthcare and education literature that says trust, a key element of collaborative practice, is forged over time through regular professional and social interactions [7, 23].

The findings did reveal that several factors influenced social interaction including the length of time nurses knew each other, interpersonal skills of nurses, and age/generational issues. Nurses reported that time could positively or negatively influence social interaction. This was not surprising given the unpredictable patient/family care demands and other workload issues nurses face on a regular basis. While this finding is not widely supported in the literature, some authors have found that a lack of time could negatively impact on the development of collaborative relationships [15, 24].

The nurses' interpersonal skills were also an influencing factor on the willingness of the nurses to socially interact. Most nurses reported they were reluctant to interact socially with other nurses who had poor attitudes and/or those who made negative comments. In addition, younger and older nurses would gravitate to nurses their own age to socially interact, and this was due to a belief that they had more in common both professionally and personally. The preference to socially interact with their own age group could be problematic given the current composition of the nursing workforce. Nurses, despite what generational background they come from, need to be able to collaborate with each other in a meaningful way in order to provide quality patient care. Differing generational attitudes towards work ethic, values, and problem solving, if not overcome, could lead to workplace conflict which in turn could lead to absenteeism and possibly turnover [25].

Nurses need time and opportunity to interact both professionally and socially for the development of their collaborative relationship. Bedwell and colleagues [26] noted that collaboration is not a one-time event but an evolving, active process whereby individuals share mutual aspirations and interests over time. Nursing leadership needs to ensure nurses regularly receive their breaks/meals by providing appropriate staffing levels and reasonable patient workload assignments, as this not only encourages social interaction, but also improves collaboration [27].

Moreover, nursing leaders should encourage social interaction through allocation of additional interaction time at program, staff, and/or professional meetings [11]. For example, staff meetings could be extended by fifteen minutes with the central purpose of facilitating informal social interaction opportunities and/or fostering a culture of collaboration among nurses. Maton et al. [28] describe this as a “deliberate action” that encourages team-building, relationship building, and the development of collaborative practice skills necessary for successful collaboration.

Our study has shown that social interaction is an important contributor of nurse-nurse collaboration. Collaboration is considered a required competency of all nurses [18, 29, 30] and is listed as one of the Healthy Work Environment standards by the American Association of Critical-Care Nurses (AACN) [12]. This standard recommends that nursing leaders address nurses who refuse to collaborate and/or exhibit poor collaborative attitudes or behaviours. Collaborative work is important to patient care and job satisfaction; nursing leaders must make it a priority to address ineffective interpersonal relationships among nurses.

An important consideration from the findings of this study is problems relating to the interpersonal skills of some of the nurses that led to a lack of interest in social interaction. This finding again highlights the importance of nursing leadership and their role in facilitating access to education programs that could improve nurses' interpersonal skills. An educational program that focuses on the development of “social intelligence” would be beneficial. Social intelligence (SI) according to Albrecht [31] is the ability to effectively interact or get along well with others and to manage social relationships in a variety of contexts. Albrecht describes SI as “people skills” that includes an awareness of social situations and a knowledge of interaction styles and strategies that can help an individual interact with others.

From the perspective of interpersonal skills, Albrecht classifies behaviour toward others as on a spectrum between “toxic effect and nourishing effect.” Toxic behaviour makes individuals feel devalued, angry, and inadequate. Nourishing behaviour makes individuals feel valued, respected, and competent. The nurses in our study reported experiencing negative comments and toxic behaviours from other nurses, and this reduced their interest in socially and professionally interacting with those nurses. Fortunately, social intelligence can be learned, first by understanding that SI encompasses a combination of skills expressed through learned behaviour and then by assessing the impact of one's own behaviour on others [31].

While it is not an easy task to be undertaken, nursing leadership needs to address the attitudes and behaviours of nurses, as these interpersonal skills are needed for both social interaction and collaboration. This could be accomplished by role modeling collaborative behaviours, having policies and/or programs in place that support a collaborative practice model, providing education on the basic concepts of SI and collaborative teamwork, and lastly facilitating the application of these concepts during social and professional interaction activities.

## 9. Limitations

The findings from this study contribute to the current limited literature on the importance of social interaction in collaborative relationships among nurses. However, there were limitations to the study. The sample size was small in quantitative terms, yet in qualitative case study design this is a characteristic rather than a limitation. The findings reflect the perspectives of oncology nurses at one organization in one geographic location, and therefore the results may limit transferability to other nursing roles and specialties in other organizations and locations. While the findings are important, we recommend further exploration of social interaction and collaboration among nurses working in different roles and clinical specialties.

## 10. Conclusions

Successful collaborative practice among oncology nurses requires the opportunity to not only work together but also socially interact. Oncology nurses working in different roles and on different clinical units reported that social interaction is important as it contributed to the development and maintenance of trust and respect in their collaborative relationships. The lack of time, age/generational differences, and poor interpersonal skills were reported as barriers to social interaction among the nurses. Nursing leadership attention to these organizational and individual factors may strengthen nurse-nurse collaborative practice and promote healthy workplaces.

## Figures and Tables

**Table 1:** Participant demographics (*n* = 14).

Variable	Category	Percentage	*N*
Gender	Male	14	2
	Female	86	12

Age	<36 years	22	3
	37–45 years	22	3
	46–55 years	36	5
	56–65 years	22	3

Highest nursing education	Diploma (equivalent to associate degree)	36	5
	Bachelor of Science	22	3
	Master of Science/Nursing	43	6

Nursing role	Staff RN	14	2
	Patient discharge coordinator	14	2
	Patient care coordinator	14	2
	Director of nursing	7	1
	Research nurse coordinator	7	1
	Clinical nurse specialist	7	1
	Nurse practitioner	14	2
	Nurse educator	7	1
	Clinical manager	14	2

Experience in current oncology nursing role	<5 years	43	6
	6–10 years	7	1
	11–15 years	14	2
	16–20 years	14	2
	>20 years	22	3

Clinical unit type	In-patient	50	7
	Out-patient (ambulatory)	50	7

Clinical disease sites	Malignant hematology (leukemia, lymphoma, myeloma)	29	4
	Allo. and auto. bone marrow transplant	14	2
	Solid tumors (head and neck, gastrointestinal, genitourinary, gynecology, prostate, lung, breast)	29	4
	Palliative care	14	2
	All disease sites	14	2
